# A149 CHARACTERIZING INAPPROPRIATE PROTON-PUMP INHIBITOR DISCONTINUATION IN PATIENTS WITH ESOPHAGEAL STRICTURES

**DOI:** 10.1093/jcag/gwac036.149

**Published:** 2023-03-07

**Authors:** K L Kecskemeti, M Borgaonkar, J McGrath

**Affiliations:** Faculty of Medicine, Memorial University of Newfoundland, St. John's, NL, Canada

## Abstract

**Background:**

Recent recommendations to reduce proton pump inhibitor (PPI) usage may cause uncertainty for clinicians and patients. We have shown increased PPI discontinuation rates in patients with esophageal strictures in recent years. Purpose: We aim to determine the appropriateness of PPI discontinuation in patients undergoing esophageal dilation for symptomatic strictures.

**Purpose:**

We aim to determine the appropriateness of PPI discontinuation in patients undergoing esophageal dilation for symptomatic strictures.

**Method:**

All patients from two gastroenterology practices who received dilations to treat symptomatic esophageal strictures in 2015-2021, (group 1: 2015-17 and group 2: 2019-21) were identified using physician billing codes for this retrospective study. Patient demographics, medications, and previous GI diagnoses were collected using endoscopy reports, nursing reports and medication records from the local hospital database. We defined *PPI discontinuation* as either a 50% dose reduction, 50% frequency reduction or complete medication discontinuation at the time of endoscopic dilation compared to the established PPI therapy. Next, we defined *inappropriate PPI discontinuation* as a patient who discontinued their PPI medication with a past history of 1. esophageal stricture, 2. Barrett’s esophagus 3. grade C/D esophagitis, or 4. experienced symptom reoccurrence after PPI discontinuation. We selected these criteria as they are consistent with both Canadian and American Gastroenterology Society GERD management guidelines. This information was coded on a standard data sheet and entered into SPSS for analysis.

**Result(s):**

In total, 223 patients were identified with an average age of 58.7 and a sex ratio of (125:98, M:F). **26 patients** discontinued their PPI medication prior to esophageal stricture dilation. The most frequent type of event was **complete PPI** discontinuation at 58% (15/26 cases). Followed by frequency reduction at 27% (7/26), dose reduction at 8% (2/26), and both dose and frequency reduction at 8% (2/26). The 26 patients with PPI discontinuations had an average length of **13 months** between PPI discontinuation and esophageal stricture dilation.

**57%** (15/26 cases) of patients meet our criteria for inappropriate PPI discontinuation (table 2). The proportion of inappropriate PPI discontinuation are as follows: Group 1: **33%** (3/9 cases) inappropriate PPI discontinuations and Group 2: **70%** (12/17 cases) inappropriate PPI discontinuations, (P=0.06) upon Chi-squared analysis.

Table 2- Inappropriate PPI discontinuations

Previous Stricture: 10/26

Barrett’s Esophagus: 0/26

Grade C/D Esophagitis: 1/26

Symptom Reoccurrence: 4/26

*Total inappropriate discontinuations*: 15/26 = 57%

*Total appropriate discontinuations (no PMH):* 11/26 = 43%

**Conclusion(s):**

Compete PPI discontinuation was the most common type of PPI discontinuation event. There was a trend toward more inappropriate PPI discontinuations in the second time period. Physicians should carefully consider indications for PPI use prior to discontinuation.

**Please acknowledge all funding agencies by checking the applicable boxes below:**

None

**Disclosure of Interest:**

K. Kecskemeti: None Declared, M. Borgaonkar Consultant of: $10K, Speakers bureau of: $20K, J. McGrath: None Declared

